# Formation of Cu_2_O Solid Solution via High-Frequency Electromagnetic Field-Assisted Ball Milling: The Reaction Mechanism

**DOI:** 10.3390/ma13030618

**Published:** 2020-01-30

**Authors:** Yingzhe Zhang, Yudao Chen, Juan Li, Wei Li, Ding Chen, Qingdong Qin

**Affiliations:** 1 College of Materials and Metallurgical Engineering, Guizhou Institute of Technology, Guiyang 550003, China; yudaochen7@gmail.com (Y.C.); 20180885@git.edu.cn (J.L.); 2 School of Energy and Power Engineering, Changsha University of Science & Technology, Changsha 410014, China; liwei11op@163.com; 3 State Key Laboratory of Advanced Design and Manufacturing for Vehicle Bodies, College of Mechanical and Vehicle Engineering, Hunan University, Changsha 410082, China; chending@hnu.edu.cn

**Keywords:** mechanical alloying, ball milling, cuprous oxide

## Abstract

The contamination of environmental water with organic pollutants poses significant challenges for society, and much effort has been directed toward the development of catalysts and methods that can decompose these pollutants. While effort has been directed toward the fabrication of Cu_2_O catalysts by ball milling, this technique can involve long preparation times and provide low yields. In this study, we synthesized a solid solution of Cu_2_O in 22 h by high-frequency electric-field-assisted ball milling below 40 °C in only one step under aqueous conditions. We investigated the catalytic activities of the produced Cu_2_O solid solution in the microwave-assisted degradation of dyes, namely rhodamine B, phenol red and methyl orange. The prepared Cu_2_O solid solution was very catalytically active and completely degraded the above-mentioned dyes within 2 min. The one-dimensional diffusion model and the phase boundary (planar) model were found to describe the kinetics well. Synergism between ball milling and the high-frequency electromagnetic field plays a key role in the preparation of Cu_2_O solid solution nanoparticles. Ball milling facilitates the relaxation of the Cu_2_O lattice and high-frequency electromagnetic radiation accelerates the diffusion of Fe atoms into the Cu_2_O crystal along the (111) crystal plane, quickly leading to the formation of a Cu_2_O solid solution.

## 1. Introduction

Industrial progress has led to water pollution that is now a serious environmental issue. The discharge of industrial wastewater is particularly problematic because wastewater may contain high concentrations of multiple non-biodegradable organic pollutants that are potentially toxic to a variety of lifeforms [1,2]. Many approaches have been proposed to alleviate the environmental pressure imposed by wastewater pollutants, including biological treatment, activated adsorption technologies, chemical treatment technologies, the use of microorganisms, the physical treatment of wastewater, and catalytic treatment [3,4,5,6]. Among these approaches, catalytic treatment is regarded to be a green and effective method for addressing the aforementioned problems [7]. Incomplete degradation of dyes and poor catalytic activity often distress researchers in the catalysis field. Microwave (MW) irradiation techniques have recently received increasing attention as means of removing refractory chemicals present in waste streams owing to advantages that include faster reaction times, better reaction selectivities, lower activation energies, reduced equipment size, lower waste production, and facile control.

Cu_2_O can decompose organic pollutants in wastewater to carbon dioxide and inorganic ions, and is considered to be one of the most promising materials for environmental catalysis applications [8]. Su et al. synthesized rhombic dodecahedral Cu_2_O nanocrystals using a facile hydrothermal method; these nanoparticles degraded aromatic organic compounds, including toluene and chlorobenzene, in a highly efficient manner [9]. Zhou et al. [10] prepared Cu_2_O particles with a variety of morphologies using a surfactant-free fabrication method; these nanoparticles were highly catalytically active toward organic pollutants. Yu et al. [11] produced Cu_2_O using a liquid-phase reduction method, and their Cu_2_O degraded more than 75% of the fluroxypyr in a sample, while the Cu_2_O particles synthesized by Ho et al. [12] exhibited excellent performance for the removal of methyl orange from aqueous media. Significant effort is currently directed toward the development and fabrication of Cu_2_O catalysts, with a variety of techniques, including hydrothermal synthesis, wet chemical methods, electrochemical methods, solvothermal synthesis, sonochemical methods, and microwave-assisted fabrication, used [13,14,15]. Ball milling, which is a mechanical chemistry method, is a green approach that involves simple experimental procedures. However, due to long preparation times and low yields, the applications of ball milling are limited. In recent years, several external field assisted ball-milling methods that involve the use of surfactants, microwaves, plasma, and ultrasound, have been investigated [16,17,18,19].

High-frequency electromagnetic-field-assisted ball milling is a new technique developed in our group [18]. This technique makes use of a non-classical energy source and has been shown to dramatically reduce processing time. Moreover, the conversion rates, product yields, and purities attained using this technique are high, and no pollutants are emitted. In addition, this technique can be used at low temperatures, thereby fostering a safe working environment. This method saves more energy and is more efficient compared to conventional heating methods, and is also more environmentally protective. Although some nanopowders have been successfully prepared, such as Cu/Fe/Fe_3_O_4_, FeOOH/Cu, and ZnFe_2_O_4_, which were prepared directly and quickly at approximately 40 °C [20], the underlying mechanisms involved in the high-frequency-assisted ball-milling synthesis technology remain unclear. The underlying formation mechanism during high-frequency- assisted ball milling is different to that of normal high-energy ball milling. In the latter process, the raw materials are cracked and become smaller and smaller in size. Under the effect of local high temperatures induced by the ball milling process, chemical reactions take place on the surfaces of some particles that are sufficiently small and have high specific surface areas, leading to nucleation. The crystal nuclei then grow progressively larger until a new phase is formed. However, because the effective collision probability during ball milling is small, the nucleation rate is very low, leading to very long preparation times. Therefore, the particles synthesized by high-energy ball milling are often too large and contain thick layers of disordered atoms (i.e., amorphous layers) on their surfaces [21,22]. Hence, elucidating the formation mechanism involved in high-frequency-assisted ball milling is of vital importance for preparing nanopowders.

It is well-known that problems associated with energy and the environment are major and difficult challenges facing society in the 21st century. To enable the widespread application of high-frequency-assisted ball milling to the production of nanopowders, research into the mechanisms and factors that govern these phase transformations is necessary. In this study, we successfully prepared Cu_2_O solid solution using this new method in only 22 h. Moreover, we revealed the underlying mechanism involved in the fabrication of Cu_2_O solid solution and also carried out a reaction kinetics study.

## 2. Experimental

**Figure 1:**
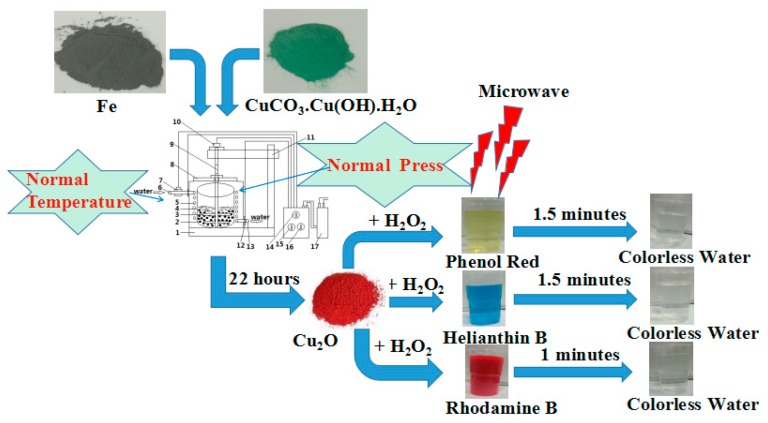
Scheme of Cu_2_O solid solution preparation and the test of its catalytic properties.

The procedure for the preparation of the Cu_2_O solid solution is shown in Figure 1. It involves the use of CuCO_3_·Cu(OH)_2_·H_2_O and Fe powder, in a 1:2 molar ratio, as the raw materials. All chemicals were of analytical grade and were used without further purification. Before the experiment, the raw materials were mixed by plate ball milling for 1 h. The mixed powder was then used in the aqueous high-frequency electromagnetic field-assisted ball milling process. The mass ratio of balls to the mixed powder was 50:1. The equipment used was designed in house, and the experimental procedure is outlined in a previous report [23]. The temperature of the solution was maintained at below 40 °C throughout the duration of the experiment. The rotation speed of the stirrer was set to 258 rpm, and the electromagnetic field was with a frequency of 200–300 kHz. After a specific time had elapsed, the milled solution was removed, filtered, and dried at 323 K for 12 h. The evolution of the transformation products was monitored by X-ray diffractometry ((XRD, dan dong tong da, Dandong, China). The particle sizes and shapes were examined by high-resolution transmission electron microscopy ((HRTEM, JEOL, Tokyo, Japan), and the concentration of the dyes in the solutions were determined by UV-Vis spectrophotometry (756 UV-Vis spectrophotometer, jinghua, Shanghai, China). 

The catalytic activities of the as-prepared samples were evaluated by examining the microwave-induced degradation of rhodamine B, phenol red and methyl orange. Microwave experiments were conducted in a microwave oven with a rated power of 700 W and a frequency of 2450 MHz. The following microwave-induced catalytic degradation process was used. Prior to microwave irradiation, Cu_2_O solid solution (0.275 mg) and 10 mL of H_2_O_2_ (30 wt%) were added into 200 mL of aqueous solution containing 15 mg/L of the dye. The suspension was stirred for 12 h in the dark in order to ensure adsorption–desorption equilibrium. The concentration of the solution at equilibrium was measured and taken as the initial concentration (C_0_). The solution was then placed into a flask that was fixed in the center of the microwave oven, after which it was irradiated with microwaves at intervals of 15 s with intervening 15-s intervals without irradiation to prevent explosive boiling. Samples of the reaction solution were removed at specific times and analyzed. The concentration (C) of the remaining dye was determined by UV-Vis spectroscopy.

In order to invest the catalyst properties of the Cu_2_O solid solution, we comparatively studied the catalytic performance of a kind of normal cuprous oxide under the same conditions. Considering that there are great differences in size, morphology and internal stress between the cuprous oxide prepared in this paper and the Cu_2_O powder purchased on the market, the normal copper oxide used in this paper was prepared by high energy ball milling in 100 mL CuCl_2_ solution using 5 g of Cu powder as the raw material. More details of the preparation process can be found in a published article [24].

## 3. Results and Discussion

### 3.1. Phase Transformation

**Figure 2:**
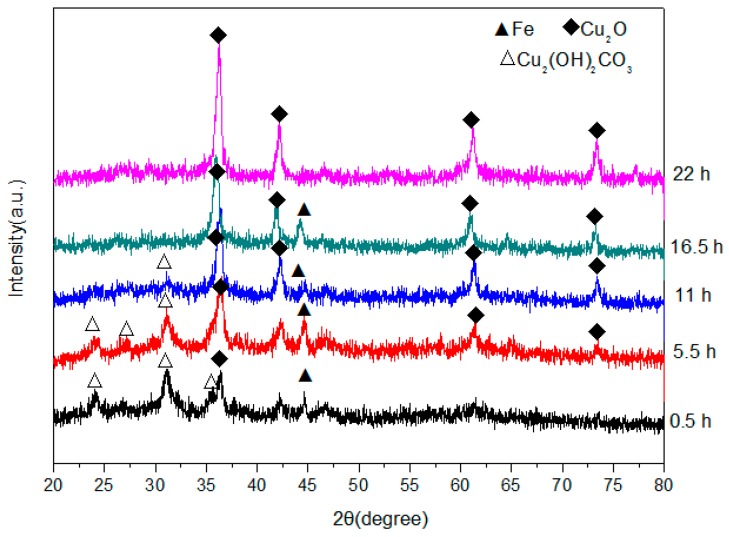
X-ray diffractometry (XRD) patterns of powders produced by high-frequency electromagnetic-field-assisted ball milling under aqueous conditions at different reaction times.

The XRD patterns of the product obtained at different elapsed ball-milling times are shown in Figure 2, which reveal that some Cu_2_O was present in the sample after only 0.5 h and that the Cu_2_O content increased with milling time. No CuCO_3_·Cu(OH)_2_·H_2_O was present after 16.5 h, and no phases were detected apart from those associated with Cu_2_O and Fe. The decomposition process for CuCO_3_·Cu(OH)_2_·H_2_O in our experiment is expressed by Equation (1):

(1) $${\mathrm{CuCO}}_{3}\cdot \mathrm{Cu}(\mathrm{OH}{)}_{2}\cdot H_{2}O+\mathrm{Fe}\overset{\begin{array}{l} \mathrm{High}-\mathrm{Frequency}\;\mathrm{Electromagnetic}\;\\ \mathrm{Field}-\mathrm{Assisted}\;\mathrm{Ball}\;\mathrm{Milling} \end{array}}{\to }{\mathrm{Cu}}_{2}O+2H_{2}O+{\mathrm{FeCO}}_{3}$$

It is known that alkaline copper carbonate begins to decompose at a temperature of 220 °C; however, the temperature of the solution in our experiment was constantly measured and remained below 40 °C at all times. This may mean that CuCO_3_·Cu(OH)_2_·H_2_O does not thermally decompose, but rather that some other special effect operates during high-frequency electromagnetic field-assisted ball milling in aqueous solution. In fact, similar phenomena were previously observed in microwave-assisted chemical reactions and were concluded to be non-thermal effects [25,26]. The authors of these studies designed a new method for microwave-assisted ball milling, in which many ferrite products with superior properties were rapidly produced [27,28]. Compared to microwave-assisted ball milling, the setup required for high-frequency electromagnetic field-assisted ball milling under aqueous conditions is simpler, the required temperature is lower, and the overall process is more energy efficient. Therefore, this method is a promising technology for the preparation of nanopowders, and may become widely adopted by industry in the future. 

The XRD patterns reveal that significant amounts of Fe powder remain in solution after 16.5 h; however, after a further 5.5 h this Fe had completely disappeared to leave only Cu_2_O, which suggests that the remaining Fe dissolved into the Cu_2_O crystal lattice to form a kind of Cu_2_O solid solution. The reaction that takes place during high-frequency-assisted ball milling in under aqueous conditions is shown in Equation (2):

(2) $${\mathrm{Cu}}_{2}O+\mathrm{Fe}\overset{\mathrm{high}-\mathrm{frequency}\;\mathrm{electromagnetic}\;\mathrm{field}+\mathrm{ball}\;\mathrm{milling}}{\to }{\mathrm{Cu}}_{2}O_{\left(\mathrm{solid}\;\mathrm{solution}\right)}$$

### 3.2. Product Microstructures and Morphologies

**Figure 3:**
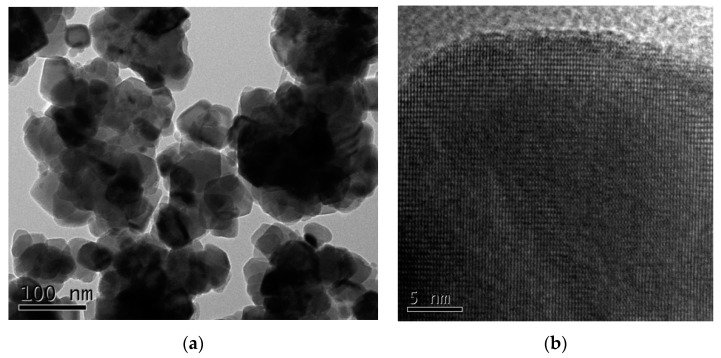
(**a**) Microstructure and (**b**) high-resolution transmission electron microscopy (HRTEM) images of the Cu2O solid solution synthesized by high-frequency electromagnetic-field-assisted ball milling under aqueous conditions.

The particle sizes and morphologies of nanomaterials determine their surface activities, specific physicochemical properties, and behavior, such Cu_2_O as adsorption, catalysis, reaction thermodynamics, kinetics, and microwave activities [29,30]. The morphologies of the prepared Cu_2_O solid solution particles were characterized by TEM, the results of which are shown in Figure 3. From the pattern it can be seen that the Cu_2_O solid solution particles are approximately spherical in shape and ranged from 10 to 50 nm in size; they also exhibit usually very large specific surface areas. Hence, the prepared Cu_2_O solid solution particles were expected to demonstrate good catalytic behavior. Furthermore, the Cu_2_O solid solution particles are well dispersed, unlike powders prepared by normal high-energy ball milling [31,32]. Good dispersion helps to keep the powder suspended in aqueous media for a long time without aggregation or settling to the bottom of the flask, resulting in fuller contact with pollutants and superior catalytic activity.

### 3.3. Catalytic Performance

**Figure 4:**
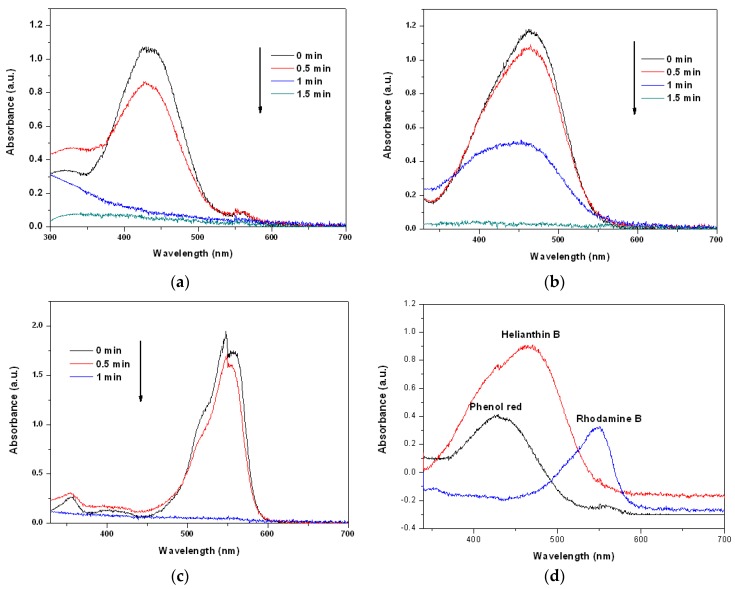
Changes of the UV-Vis spectra of (**a**) phenol red, (**b**) helianthin B, and (**c**) rhodamine B solutions after microwave irradiation in the presence of Cu_2_O solid solution nanoparticles for different periods of time, as well as (**d**) all the phenol red helianthin B and rhodamine B degraded by normal Cu_2_O by microwave irradiation for 5 min.

We carried out MW-assisted degradation experiments in this study. Figure 4a–c show the degradation of dye solutions containing phenol red, helianthin B, and rhodamine B separately when exposed to MW radiation, as functions of time, using the prepared Cu_2_O solid solution as the catalyst. The acquired spectra reveal that all dyes were degraded completely in less than 2 min. Clearly, the Cu_2_O solid solution synthesized in this study can catalyze the degradation of many types of organic dye quickly and thoroughly, suggesting that the Cu_2_O solid solution prepared in this study is very clearly advantageous and has broad application prospects. Figure 4d shows UV-Vis spectra of all the phenol red, helianthin B and rhodamine B degraded by normal Cu_2_O by microwave irradiation for 5 min. From the patterns it can be found that even the degradation time last for 5 min much of the organic pollutants were left. It may suggest that the Cu_2_O solid solution synthesized by high frequency assisted ball milling own much better catalytic degradation performance than the normal cuprous oxide prepared by high energy ball milling, which may be caused by the lattice stress of cuprous oxide caused by the effect of solid solution.

Microwaves are regarded as a special kind of energy and, to date, numerous mechanisms have been proposed to explain the synergistic effect of microwave radiation and catalysts. Generally, most researchers believe that hot spots created on the surfaces of the reagents through interactions between microwaves and microwave-absorbing materials play major roles in the degradation of dyes [33]. The enhanced catalytic properties observed in the present study may originate from the special structure of Cu_2_O solid solution. The synthesized Cu_2_O solid solution particles have nanoscale dimensions, and their specific surface areas are sufficiently large to absorb significant amounts of dye. Cu_2_O solid solution is also known as a material with excellent microwave-absorbing properties. Furthermore, the milled particles are usually irregular and, as a consequence, the electrons are distributed unevenly. This non-uniform distribution of electrons results in the formation of a large number of dipoles with good microwave-absorbing properties [34,35]. In the catalysis experiment, the combination of microwaves and Cu_2_O solid solution produce a large number of “hot spots” on the surfaces of the irregular Cu_2_O solid solution particles, where more rapid oxidation and combustion of the organic pollutant molecules take place, leading to highly efficient degradation as a consequence.

### 3.4. Formation Mechanism and Reaction Kinetics

#### 3.4.1. Solid-State Formation of Cuprous Oxide

**Figure 5:**
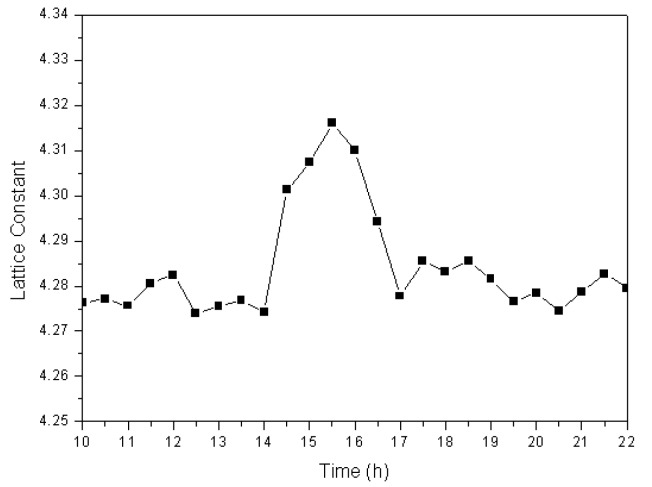
Lattice constant of the Cu_2_O solid solution as a function of time.

Phase-transformation analyses revealed that, by combining of functions of ball milling and a high-frequency electric field, large amounts of Fe completely dissolve into the newly formed cuprous oxide to produce a Cu_2_O solid solution. To determine the mechanism for the formation of cuprous oxide, it is necessary to investigate variations in the lattice constant of the prepared Cu_2_O solid solution at different times during the formation process. We used the XRD data and the Jade 6.0 software to calculate the lattice constant at different times, the results of which are shown in Figure 5. The lattice constant was observed to remain at approximately 4.275 over the first 14 h. During this period, the reaction described in Equation (1) takes place. However, the lattice constant increased quickly at 14.5 h, and stayed at approximately 4.30 for the next 2.5 h. Many iron atoms become embedded in the cuprous oxide lattice during ball milling in the high-frequency electric field, which results in lattice relaxation; therefore the Cu_2_O solid solution lattice constant increases. By the 17th hour of the experiment, the lattice constant was observed to have decreased to approximately 4.280. At this time, all the Fe had dissolved into the Cu_2_O lattice to become interstitial or replacement atoms, and Cu_2_O solid solution formed.

**Figure 6:**
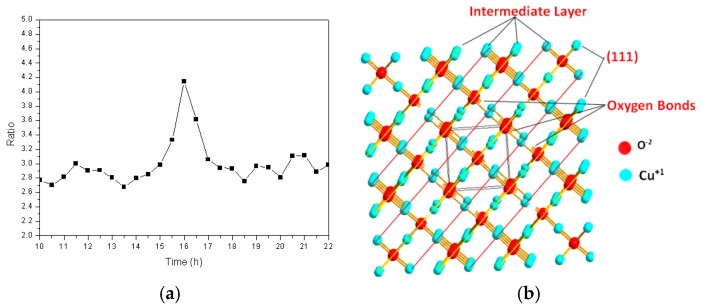
(**a**) The ratio of the intensity of the (111) crystal plane diffraction peak to that of the (200) crystal plane as a function of time; (**b**) Atomic arrangement of the cuprous oxide lattice.

In order to further study the insertion pathways of the iron atoms during the formation of the cuprous oxide solid solution, the intensity ratio of the diffraction peak corresponding to the (111) crystal plane to that of the (200) crystal plane was calculated at different times, the results of which are shown in Figure 6a. This ratio was approximately 2.8 over the first 14 h, after which it increased quickly, by almost a factor of 1.5 (to 4.1), suggesting that grains grow rapidly in the (111) direction. The (111) plane of the cuprous oxide lattice formed an atomically dense and arranged surface that was prone to sliding, which exposed the outermost regions of the particles. Figure 6b shows the atomic arrangement of the cuprous oxide lattice. Many unsaturated oxygen bonds are observed in the (111) crystal plane, and many of these are exposed to the surface exterior. It is known that iron atoms are easily reduced, and monovalent oxygen atoms are strongly oxidizing. The two outermost iron electrons are activated and easily bond to the exposed unsaturated oxygen atoms of different particles in a high-frequency electromagnetic field. In turn, this results in the lengthening of the powder particles in the (111) plane. By the 17th hour, the diffraction peak intensity ratio returned to its original value, which is possibly ascribable to the Bracken effect during ball milling, in which elongated grains are crushed into their original spherical shapes.

#### 3.4.2. Reaction Kinetics 

**Figure 7:**
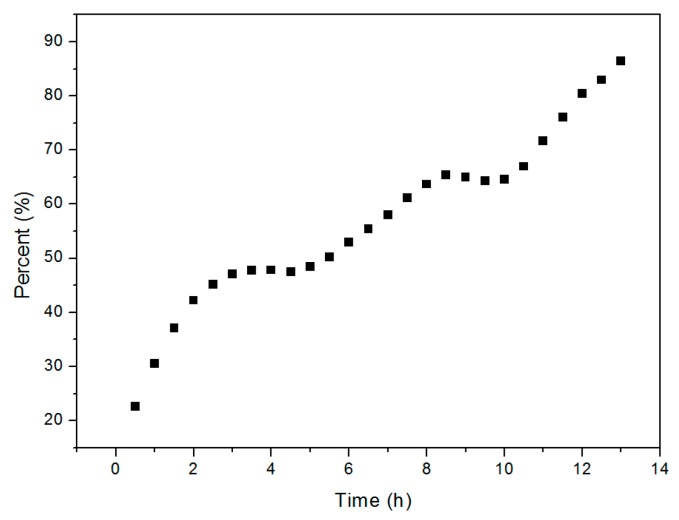
Percentage of Cu_2_O solid solution formed as a function of reaction time.

The kinetics of the Cu_2_O solid solution formation reaction were studied in order to gain additional insight into the reaction mechanism. Each of the phases formed during the reaction process and the quantity (%) of Cu_2_O solid solution formed by the process over time were determined by XRD. As reported, XRD is a common method for estimating the percentage content of crystal phase [36,37]. In this paper, the raw material powders as well as the products were always crushed by ball milling in solution. So that it is reasonable that there is no texture phenomenon and it is reasonable to calculate the percentage content of cuprous oxide according to the test results of XRD. As we know, the content of the phase is directly related to the peak height of the XRD diffraction peak. The higher the peak height is, the higher the content of the phase is. The highest peak belongs to (111) crystal plane that is at the position of 36.41 degree dispersion angle of XRD of Cu_2_O and (20-1) crystal plane that is at the position of 31.29 degree dispersion angle of XRD of Cu_2_(OH)_2_CO_3_ was used to estimate their content, respectively. More details of the calculation method can be found in the relevant literature reports [38,39]. The time-evolution of the Cu_2_O solid solution percentage determined from the XRD pattern is shown in Figure 7, which shows that the transformation involves two steps that are apparent at the fifth and tenth hours. This suggests that different factors dominate during the three stages of high-frequency electromagnetic-field-assisted ball milling.

As a mechanochemical reaction, the kinetics of high-frequency electric-field-assisted ball milling can be evaluated by fitting data to a regular solid-state reaction model, which is expressed by:

*F*(*a*) = *kt* (3)

where *F*(*α*) describes the dependence of the reaction rate on the process mechanism, *α* is the fraction of the target product, which ranges from 0 at the beginning of the reaction to 1 at completion, *k* is the rate constant, and *t* is the reaction time. Various models that describe the kinetics of solid-state reactions have been proposed in the literature, and some of the most common ones are listed in Table 1. 

These mechanisms are based on different geometrical assumptions for the shapes of the particles (spherical, cylindrical, and planar) and various driving forces (interfacial growth, diffusion, nucleation, and growth of nuclei) [40,41]. 

Relevant experimental data for the high-frequency-assisted ball milling reaction were substituted into Equation (3); the relationships between the reaction time *t* and the fraction *α*, and the associated coefficients of determination (R^2^) for the various models are listed in the Table 1. The rate constants *k* are easily obtained from the line of best fit, and the values of *t* = *F*(*1*)/*k*, which provide an estimate of the time required for the chemical reaction to reach completion, are also listed in Table 1. The table reveals that the R^2^ values for the one-dimensional diffusion, phase boundary (planar), phase boundary (cylindrical), Nucleation and growth (Avrami) and Nucleation and growth (Erofeev) models all exceed 0.94. As showed in Figure 2, it can be seen that the experimentally determined time for the raw materials to be completely converted into the target product (Cu_2_O solid solution) is about 22 h. However, the theoretical time calculated for the reaction to complete by the phase boundary (cylindrical) model, Nucleation and growth (Avrami) model and Nucleation and growth (Erofeev) models are 32.89 h, 71.45 h and 66.88 h respectively, those all are much longer than the experimentally determined time Therefore, we conclude that the one-dimensional diffusion and phase boundary (planar) models by which the theoretical time for the reaction are 23.06 h and 25.46 h respectively could best describe the dynamics of the reaction process.

During the high-frequency-assisted ball milling preparation of Cu_2_O solid solution, the raw materials first crack into smaller particles by the impact of the balls. As the milling process progresses, the raw materials become smaller in size and the specific surface area increases, which enhances chemical activity. Under the combined effects of ball milling and the applied high-frequency electric field, the reaction described in Equation (1) takes place to form Cu_2_O. Fe^2+^ is also generated during this process, which then diffuses into the Cu_2_O crystal lattice. At this stage, one-dimensional diffusion plays a major role. By the 14th hour, all of the CuCO_3_·Cu(OH)_2_·H_2_O had reacted with Fe; however some Fe still remained. The reaction between Cu_2_O and Fe through the phase-boundary mechanism subsequently plays the leading role. 

## 4. Conclusions

In this manuscript, Cu_2_O solid solution powder was produced in only 22 h at below 40 °C by high-frequency electromagnetic-field-assisted ball milling by only one step. The well-dispersed Cu_2_O solid solution particles were all in nanosize and they exhibited strong catalytic activities towards phenol red, helianthin B, and rhodamine B, which were all completely degraded in less than 2 min. The high catalytic activities observed during the microwave-induced catalytic degradation process are ascribable to the combined effects of the special structures of the synthesized Cu_2_O solid solution particles and microwave radiation. 

In this study, we also determined that the phase boundary (planar) model and the one-dimensional diffusion model more accurately represent the kinetics for the formation of Cu_2_O solid solution. During ball milling and in a high-frequency electric field, the reaction between CuCO_3_·Cu(OH)_2_·H_2_O and Fe progresses and Cu_2_O is formed. At the same time, the produced Fe^2+^ diffuses into the Cu_2_O crystal lattice structure. After a further 14 h, almost all of the CuCO_3_·Cu(OH)_2_·H_2_O had disappeared, and the remaining Fe reacts with the cuprous oxide on its (111) crystal plane to form a solid solution of cuprous oxide. 

Compared to microwave-assisted ball milling, the setup required for high-frequency electromagnetic-field-assisted ball milling under aqueous conditions is simpler, the required temperature is lower, and the process is overall more energy efficient. Therefore, it is a promising method for the preparation of nanopowders that may become widely adopted by industry in the future. 

## Figures and Tables

**Table 1:** Kinetics models usually employed for solid-state reactions.

Model	*F*(*a*) = *kt*	K	R^2^	Reaction Time
One-dimensional diffusion	*a*^2^ = *kt*	0.04336	0.94	23.06 h
Two-dimensional diffusion	(1 − *a*) × ln(1 − *a*) + *a* = *kt*	0.03329	0.91	30.04 h
Three-dimensional diffusion (Jander)	(1 − (1 − *a*)^1/3^))^2^ = *kt*	0.01217	0.85	80.53 h
Three-dimensional diffusion (Ginstling–Brounshtein)	1 − 2/3 × *a* − (1 − *a*)^2/3^ = *kt*	0.00874	0.89	38.13 h
Phase boundary (planar)	*a* = *kt*	0.03928	0.95	25.46 h
Phase boundary (cylindrical)	1 − (1 − *a*)^1/2^ = *kt*	0.03037	0.94	32.89 h
Phase boundary (spherical)	1 − (1 − *a*)^1/3^ = *kt*	0.02355	0.93	42.04 h
Nucleation and growth (Avrami)	((−ln(1 − *a*))^1/2^ = *kt*	0.05202	0.94	71.45 h
Nucleation and growth (Erofeev)	((−ln(1 − *a*))^1/3^ = *kt*	0.03588	0.94	66.88 h
Nucleation and growth (Avrami–Erofeev)	[−ln(1 − *a*)]1/*m* = *kt*, 0.5 ≤ *m* ≤ 4	0.02842	0.93	67.84 h
1-D nucleation and constant growth rate	ln *a* = *kt*	0.07575	0.88	−1.32 × 10^−5^ h
Random nucleation and rapid growth	−ln(1 − *a*) = *kt*	0.10478	0.89	131.85 h
Chemical reaction (C1.5)	(1 − *a*)^−1/2^ − 1 = *kt*	0.08924	0.81	−11.21 h
Chemical reaction (C2)	1/(1 − *a*) − 1 = *kt*	0.316	0.71	3,164,553.80 h
